# Use of Intervention Mapping for Occupational Risk Prevention and Health Promotion: A Systematic Review of Literature

**DOI:** 10.3390/ijerph18041775

**Published:** 2021-02-11

**Authors:** Maartje C. Bakhuys Roozeboom, Noortje M. Wiezer, Cécile R. L. Boot, Paulien M. Bongers, Roosmarijn M. C. Schelvis

**Affiliations:** 1 Department of Healthy Living, Netherlands Organisation for Applied Scientific Research (TNO), Schipholweg 77, 2316 ZL Leiden, The Netherlands; noortje.wiezer@tno.nl (N.M.W.); paulien.bongers@tno.nl (P.M.B.); 2 Department of Public and Occupational Health, Amsterdam Public Health Research Institute, VU University, Amsterdam UMC, Van der Boechorststraat 7, 1081 BT Amsterdam, The Netherlands; crl.boot@amsterdamumc.nl; 3 HR Department, Erasmus University, P.O. Box 1738/3000 DR Rotterdam, The Netherlands; roos.schelvis@eur.nl

**Keywords:** intervention mapping, occupational risk prevention, occupational health promotion, interventions

## Abstract

*Aim:* Intervention mapping (IM) is a method to systematically design interventions that is applied regularly within the public health domain. This study investigates whether IM is effectively used within the occupational safety and health domain as well. Specifically, this study explores the relation between the fidelity regarding the use of the IM protocol for intervention development, the implementation process and the effectiveness of the occupational risk prevention and health promotion interventions. *Methods:* A systematic review was conducted including articles on development, implementation, and effects of occupational risk prevention and health promotion interventions that were developed according to the IM-protocol. By means of a checklist, two authors reviewed the articles and rated them on several indicators regarding the fidelity of the IM-protocol, the implementation process, and the intervention effect. *Results:* A literature search resulted in a total of 12 interventions as described in 38 articles. The fidelity to the IM-protocol was relatively low for participation throughout the development process and implementation planning. No relationship was found between fidelity of the IM-protocol and the intervention effect. A theory-based approach (as one of the core elements of IM) appears to be positively related to a successful implementation process. *Conclusion:* Results of the review suggest that organizing a participative approach and implementation planning is difficult in practice. In addition, results imply that conducting matrices of change objectives as part of the intervention development, although challenging and time-consuming, may ultimately pay off, resulting in a tailored intervention that matches the target group.

## 1. Introduction

Exposure of employees to safety and health risks at work is a major problem. Of all persons aged 15–64 that work or have worked previously, 7.9% reported a work-related health problem in the preceding year [1]. In 2016, in 3182 fatal accidents were reported in the EU [1]. These numbers illustrate the urgency for effective occupational risk prevention and health promotion interventions (ORP-HP interventions).

Despite the need for these types of interventions, meta-analyses show they often do not sort the intended effects [2,3,4]. When an intervention is not effective, there are several explanations. Either the intervention was based on incorrect theoretical assumptions, the intervention did not consist of the effective ingredients to accomplish behavioral change on the intended outcomes, or the intervention was not implemented successfully (or a combination of the above). 

ORP-HP interventions often entail multiple components and aim for behavioral change at different levels of the organization [5]. Due to this complexity, developing effective and successfully implemented ORP-HP interventions is difficult. According to the Medical Research Council, complex interventions are especially at risk for failure due to implementation problems [5]. This implies that the development and implementation of ORP-HP interventions ask for a thorough approach, with an explicit focus on the implementation process.

Different frameworks have been used for intervention development, e.g., the RE-AIM model [6], the Behavior Change Wheel [7], and the PRECEDE-PROCEED model [8]. Another well-known framework for health program planning is intervention mapping (IM), a systematic planning protocol for the development of behavioral change interventions [9] that is well adopted within the general health domain [10,11]. 

IM consists of six steps as described by Bartholomew et al. [9] (see Table 1). In Step 1, a needs assessment is conducted to identify the target behavior and behavioral and environmental determinants that need to be changed. In Step 2, the program objective is formulated and performance objectives are identified (specific behavioral actions needed to reach the program objective). To target the performance objectives, determinants are identified for each performance objective. By crossing performance objectives with behavioral determinants, matrices of change objectives are created. In Step 3, theory-based intervention methods are selected that target the determinants and help achieve the change objectives and translated into strategies or applications. In Step 4, the strategies are integrated into an intervention program. In Step 5 the implementation of the intervention program is planned. In the sixth step the process and effect evaluation are planned. 

There are four characteristics of IM that seem to make IM particularly appropriate for developing (complex) ORP-HP interventions. The first is the theory and evidence based approach [9,12], which encourages explicit use of theory and empirical evidence in defining the problem, the intended behavioral changes, and the mechanism to achieve these changes by making a logic model of the problem, conducting matrices of change objectives and choosing theory and evidence-based change methods. The aim is to ensure that the intervention is targeted at the right determinants and that the intervention contains effective ingredients for the intended behavior changes.

The second characteristic is the participative approach [9,12] that encourages stakeholder involvement in decision-making by forming a working group at the start of the project with different stakeholders, e.g., workers, managers, HR, experts, policymakers, and involving relevant stakeholders in all phases of intervention and implementation planning. The aim of this approach is to ensure that the intervention fits in with the needs of the target group, the implementors, and the context of the organization.

The third characteristic is the ecological approach [9,12], which considers the complex and layered context in which the intervention is developed and implemented (by considering behavior as well as environmental factors and targeting both with the intervention). To accomplish behavior change at the worker level, the intervention often has to target the broader context of the organization or different actors in the organization (e.g., the employer). The aim of the ecological approach is to ensure that the intended behavior changes are supported by the different layers of the organizational context.

The fourth characteristic of IM is that implementation planning of the intervention is part of the intervention development [13]. In the last decade, the focus on the implementation of interventions has emerged rapidly, providing various implementation frameworks. However, despite the increasing attention for implementation, in practice, the planning of implementation strategies often starts after the intervention has already been developed. Planning the implementation process in the intervention development phase may decrease the risk of unsuccessful implementation. 

Because of these characteristics, IM appears to be an appropriate method for the development and implementation of ORP-HP interventions [12]. Recently, Fassier et al. [14] have systematically reviewed the fidelity (the extent to which the IM-steps are followed according to protocol) of the use of the IM protocol and the effects of the interventions in work disability prevention. Out of eight studies included, two were reported as effective and one as partially effective. The authors link the low number of effective interventions to issues in relation to the fidelity of the intervention development according to the IM protocol. However, issues in relation to implementation were not taken into account in their study.

In this study, we will systematically review ORP-HP interventions on the fidelity of the application of the IM protocol and their effects. Additionally, we add to this review information on the implementation process to get more insight into the occurrence of implementation issues. The objective of this study is to explore the relationship between intervention development, implementation process, and intervention effects. Based on this objective, the following research questions were formulated:

1. What is the fidelity of the use of the intervention mapping protocol regarding the core IM characteristics (participation, theory-based approach, ecological approach, implementation planning)?
2. To what extent are interventions developed following the IM protocol successfully implemented?
3. To what extent are interventions developed following the IM protocol effective?
4. Is the level of fidelity to the IM-protocol related to the implementation success and to the effectiveness of the interventions?

## 2. Methods

### 2.1. Literature Search

The selection criteria for study inclusion were based on the study objective. Studies on ORP-HP interventions developed by IM were included, that described intervention design, effect evaluation, and process evaluation. We specifically searched for intervention studies of which the intervention design, and the process and effect evaluation were published. This called for a semi-systematic approach, focusing on selecting intervention design articles in the first step, and searching for corresponding effect and process evaluation articles in the second step. 

First, a search was conducted in the database of intervention mapping (www.interventionmapping.com/references) with the search term “work” (25-07-2019). This database consists of 1000 references of peer-reviewed published articles that use IM. All titles matching the search term “work” were reviewed to identify articles using the following inclusion criteria: (1) description of the development of ORP-HP interventions; (2) explicit use of the IM protocol. ORP-HP interventions were defined as interventions aimed at workers, to prevent them from work-related illness, accidents or injuries, or promote their health and wellbeing. Excluded were interventions aimed at tertiary prevention (return to work). Additional searches were carried out in PubMed and Scopus with search terms ‘intervention mapping’ and ‘occupational’ and/or ‘risk prevention’ and/or ‘work’ and/or ‘intervention’, to check for any other IM design articles in the occupational domain that could be included.

An additional search was conducted to find effect and process evaluation articles of the studies of the included articles on intervention development. These articles were identified by searching reference lists of included articles and by specifically searching for other articles from authors of the design articles.

### 2.2. Data Extraction and Synthesis

To review the fidelity of the intervention development according to the IM protocol, an IM fidelity checklist was developed that contained a list of 13 items that corresponded to a large extent to the activities of the IM steps (see Table 1), extracted from the third edition of the Intervention Mapping textbook [9] and crosschecked with the checklist of Fassier et al. [14]. The checklist contains the activities of the IM protocol that relate to the core characteristics of IM (participation, theory-based approach, ecological approach, and implementation planning). Step 6 was not included in the checklist since the planning of the evaluation was not hypothesized to be related to the implementation process or the intervention effects. Two authors (MBR and RS) rated each activity as either + (executed) or +/− “partially executed”, or − “not executed (or not measured/described)”. 

To review the implementation process, a process implementation checklist was developed. Since in general, the operationalization of process indicators differs substantially between articles, this checklist was used to rate the process indicators in a comparable manner. The checklist was based on the commonly used Steckler and Linnan framework for process evaluation [15], including reach, dose delivered, dose received, and fidelity. “Satisfaction” was added to gain extra information on the satisfaction and acceptance of the intervention by the target group. The process indicators were rated based on the data as presented in the articles, using the evaluation checklist. Two authors (MBR and RS) rated the implementation components as either ++ (excellent), + (satisfactory), +/− (moderate), or − (unsatisfactory).

To review the effects of the interventions based on the effect evaluations, two authors (MBR and RS) rated the interventions as either ++ (all primary and secondary outcomes effective), + (all primary outcomes effective and secondary outcomes partially or not effective), or +/− (primary and/or secondary outcomes partially effective (but not all primary outcomes effective)) or − (all primary and secondary outcomes not effective).

To develop the checklists and rate the articles, first, rating criteria were chosen based on the literature and expert opinions of the research team. Second, the checklists were tested by means of a pilot evaluation with two articles by two authors (MBR and RS), results were discussed, and the checklist was adjusted. In addition, half of the articles were rated by two authors (MBR and RS), scores were compared and discussed, and the checklist was finalized. Then all articles were rated by two authors, and disagreements were discussed until consensus was reached. The checklists with rating criteria can be found in Table S1.

### 2.3. Analyses

After the IM fidelity of the intervention development, the implementation process and the intervention effects were reviewed and rated with either ++, +, +/−, or –, each rating was quantified by scoring ++ = 3 (process indicators and effects), + = 2, +/− = 1, and − = 0. In addition, means were calculated (if no more than half of the scores was missing) for the fidelity of the activities related to participation, theory-based approach, ecological approach, and implementation planning, as well as for the overall IM fidelity (step 1a−step 5d), the implementation process and the intervention effects. Scatterplots were built in Excel to visually map the relation between the IM fidelity, the implementation process, and the intervention effects.

## 3. Results

### 3.1. Included Articles

**Figure 1:**
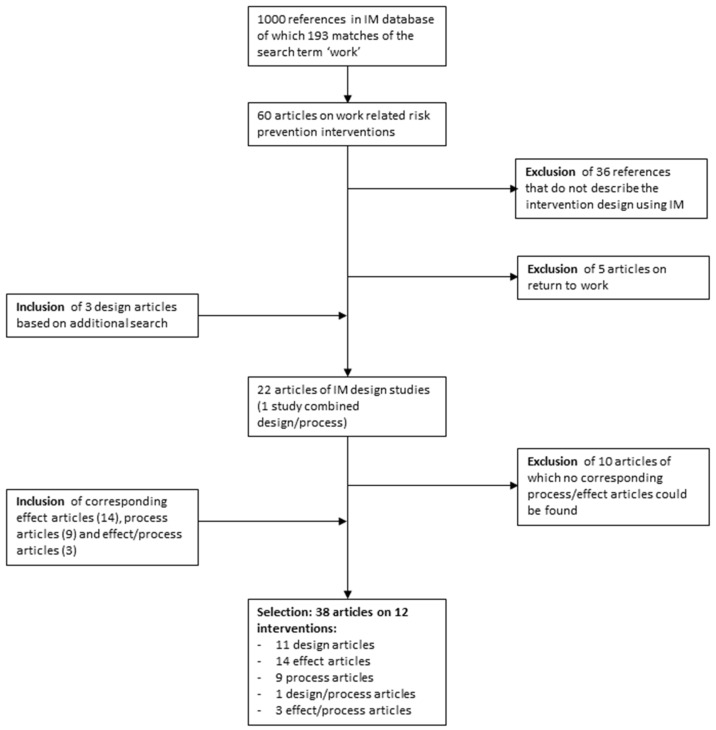
Flow-chart of included studies.

A search in the database of Intervention Mapping resulted in 193 ‘matches’ (i.e., the term “work” could be used several times in the same article) (Figure 1). In the next step, articles were identified on ORP-HP interventions, resulting in 60 records. In the following step, articles were excluded that do not describe the development of the intervention (exclusion of 36 articles). In the next step, articles were excluded that focused on tertiary occupational risk prevention (5 articles are excluded). In addition, three unique design articles were selected based on an additional search in PubMed and Scopus. This procedure led to the inclusion of 22 articles describing the intervention development (study design articles). 

For each of the design articles included at this point, a search was carried out to find process and effect evaluation articles on the interventions as described in the design articles (by performing a search based on (co)authors names and the name of the intervention). The design articles for which a published process and effect evaluation could not be found were excluded (10 articles excluded). For each of the remaining design articles, the process and effect evaluation were included, resulting in a total of 38 articles (11 design articles, 14 effect articles, 9 process articles, 1 article combining the intervention design and the process evaluation, and 3 articles combining the effect and process evaluation) on 12 interventions.

The included studies and the characteristics of the interventions are summarized in Table S2. Eight of the interventions were aimed at (amongst others) weight gain prevention and/or physical activity promotion in the workplace [16,17,18,19,20,21,22,23], two studies focused on influenza vaccination of workers [24,25], one intervention aimed at workers’ safety [26], one intervention focused on the reduction of quartz exposure [27]. Three interventions focused on (amongst others) mental-health-related outcomes, e.g., workability [22], need for recovery and relaxation [21], work engagement, and mental health [20]. The interventions covered a variety of sectors, and some were targeted at specific sectors: construction sector [18,22,27], health care [17,24,25], metal industry [26], financial service sector [21], and research institutes [20].

### 3.2. Intervention Design According to IM Protocol

Results of the fidelity review can be found in Table 2 for each of the IM (sub)steps (see Table S3 for more detail). The scores that are used for the figures can be found in Table S6. Results are reported below in relation to the core IM characteristics: participation, theory-based approach, ecological approach, and implementation planning. 

### 3.3. Participation

The first step of IM is to compose a participatory group of stakeholders (planning group) that is involved in all steps of the intervention design. Only two of the studies explicitly mentioned the formation of a planning group (step 1), however, most of the studies involved the target group, implementers, or other stakeholders at different phases of the process. In eight of the studies, the target group and other stakeholders participated during Step 1 and/or Step 2. In four of the studies, either the target group or other stakeholders participated in these steps. The majority of the studies (7 studies) involved the target group in the design of the intervention program (Step 3 and/or 4). In three studies, the target group was not involved directly, but other stakeholders participated in the design of the intervention program. In nine of the studies, the implementors were involved in the implementation planning (Step 5).

### 3.4. Theory Based

All studies conducted a needs assessment, and all but one of the studies mentioned causal pathways to describe the logic model of the problem (step 1). Five of the studies constructed matrices of change objectives (step 2). All of the studies chose theory- and evidence-based change methods (step 3). 

### 3.5. Ecological Approach

Most of the studies (10 studies) differentiated between behavioral and environmental factors in conducting a logic model of change (step 3). All studies included in their interventions both components that targeted the worker as well as environmental context (e.g., the workplace) (step 4). Table S2 provides an overview of the interventions and program components.

### 3.6. Implementation Planning

Only one of the studies explicitly identified all potential program users: adopters, implementers, and maintainers. All other studies identify adopters and implementers but did not identify maintainers. Only one of the studies explicitly formulated performance objectives for program use. However, seven studies identified drivers and barriers for implementation, and almost all of the studies (11 studies) designed interventions for implementing the intervention program, e.g., by developing manuals, protocols, communication plans, or taking other measures to ensure the fidelity and overcoming anticipated barriers for implementation. 

### 3.7. Overall IM Fidelity

**Figure 2:**
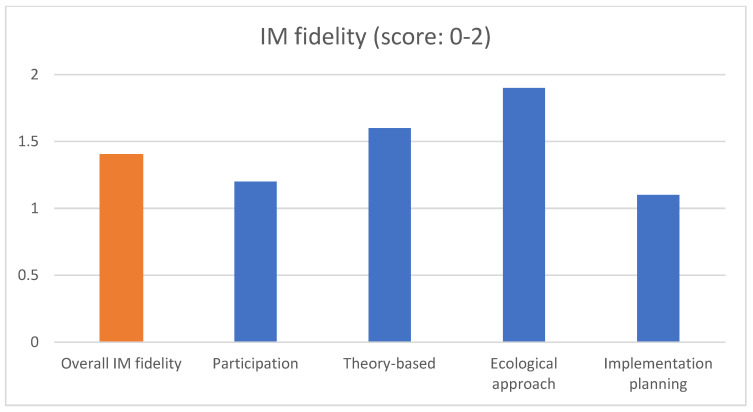
Overall IM fidelity (step 1a–5d) and fidelity of IM characteristics.

Of the core IM characteristics, the fidelity of IM activities related to the ecological approach was highest, followed by the fidelity of activities related to the theory-based approach (see Figure 2). The fidelity of activities related to participation and implementation planning was considerably lower. The low score on participation was mainly due to a lack of a planning group in most of the studies. The low score on implementation planning was due to a lack of identifying implementors and maintainers as potential program users and a lack of specifying outcomes and performance objectives for program use.

### 3.8. Evaluation of Implementation Process

Of the eight studies that calculated reach as a proportion of the participating workers, three studies reported an excellent reach (++), and five reported an unsatisfactory reach (−). Of the eight studies that reported information on the dose delivered, six reported an excellent dose delivered (++), and two reported a moderate dose delivered (+/−). All but one study provided information on the dose received. Only one of these studies reported an excellent dose received (++), seven reported a moderate dose received (+/−), and three reported an unsatisfactory dose received (−). Information on the fidelity was reported in eight of the studies, and only one reported satisfactory fidelity (+). Ten studies reported information in relation to participants’ satisfaction with the intervention. One of the studies reported an excellent satisfaction (++), eight reported a satisfactory satisfaction (+), and one study reported a moderate satisfaction (+/−). More detailed information on the review of the implementation process can be found in Table S4a,b.

### 3.9. Evaluation of Effects of the Intervention

Six studies found the intervention to be effective in changing primary outcomes. Two of these studies reported significant changes in both primary and secondary outcomes, whereas four of these studies reported changes for the primary outcomes only. Three studies were found to be partially effective, and four as not effective. More detailed information on the review of intervention effects can be found in Table S5.

### 3.10. Relation between Intervention Design, Implementation, and Effect

There appear to be no clear associations between either the overall IM fidelity and the implementation process (see Figure 1 in Figure S1) or the overall IM fidelity and the intervention effects (see Figure 2 in Figure S2).

**Figure 3:**
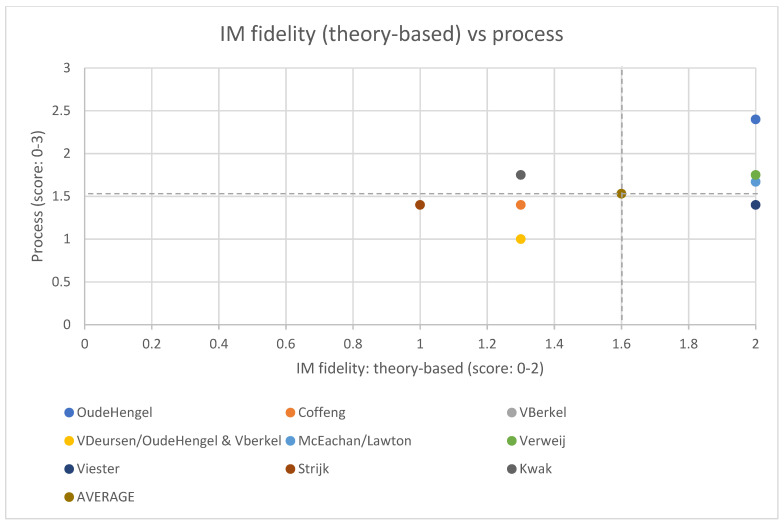
Scores of the fidelity of the theory-based approach (score: 0–2) and the implementation process (score: 0–3) per study (the dotted lines show the average scores for the IM fidelity (theory-based approach) and the implementation process). For three of the process evaluations, there was not enough data available to calculate a process score [25,50,54].

Comparing fidelity scores of the core IM characteristics with the implementation process and intervention effects, there only appears to be an association between the fidelity of IM activities related to the theory-based approach and the implementation process (Figure 3). A high score on the fidelity of IM activities related to the theory-based approach, appears to be associated with a high score on the implementation process. For none of the other core IM characteristics, the fidelity appears to be associated with either the implementation process or the intervention effects.

## 4. Discussion

The aim of this article was to explore the relationship between the fidelity regarding the use of the IM protocol in intervention development, implementation and effects of the ORP-HP interventions. First, this study investigated the fidelity of the use of the IM protocol for ORP-HP intervention development. Subsequently, this study investigated to what extent ORP-HP interventions developed following the IM protocol are successfully implemented and effective, and whether the level of fidelity to the IM-protocol is related to implementation success and intervention effects.

### 4.1. Fidelity of the IM Protocol

Participation is considered an important aspect of the development, implementation and evaluation of ORP-HP interventions [55] to ensure that the intervention fits in with the needs of the target group (increasing the support base) and the context of the organization (ensuring the feasibility of the intervention activities) [56]. However, consistent with the findings of Fassier et al. [14] and Bouché et al. [11] the included studies did not follow all steps of the participative approach as described in the IM-protocol. Of all the included studies, only two explicitly reported the formation of a working group including relevant stakeholders (e.g., target group, management, supervisors, policymakers, experts, implementors) at the start of the project. Although in most studies the target group, implementers, or other stakeholders were involved during different phases of the process, five of the studies did not involve the target group in the intervention design [16,17,22,25,27]. Especially in this step, the participation of the target group is important to ensure the intervention design is suitable for the potential users [9]. In their discussions, several authors of the included studies stress the importance of the involvement of all stakeholders during the entire process of intervention design and implementation and consider the lack of support of different stakeholders (especially from management) during implementation as an important barrier for the implementation success of their interventions. Four studies recommended to further improve participation of all layers of the organization for different reasons, to raise support from employees and management [33,36,46], to investigate preconditions for intervention success [36], and to use perspectives of the target group when choosing methods to deliver the intervention [49].

The theory-based approach is another core characteristic of IM is [9,12], to ensure that the intervention is targeted at the right determinants and the intervention contains effective ingredients for the intended behavior changes. The theory-based approach of IM prescribes the use of theory and empirical evidence by making a logic model of the problem, conducting matrices of change objectives, and choosing theory and evidence-based change methods. This study shows that the included studies had difficulties following all the detailed steps of the theory-based approach. Although all included studies conducted a logic model of the problem, and selected theory and evidence-based change methods, in contrary to the findings of Fassier et al. [14], the majority of the studies did not develop matrices of change objectives. This may imply that important sub-steps to make a theory based logic model of change are missed, because the matrices of change objectives help specify the behaviors the intervention actually has to target. One of the reasons, as mentioned by Kwak et al. [16] for not constructing a matrix of change objectives, is that the program outcomes involve several different behaviors, making matrices of change objectives (too) complex, extensive, and time-consuming [16]. Studies that did conduct matrices of change objectives, also commented in their discussion that it was a very time-consuming effort, and not always feasible in relation to planning and budget [18,19,22]. In addition, the studies differ in the level of detail they present regarding the information on why and how choices were made for the particular change strategies, tools, and materials (step 3). This is remarkable since, in this step of IM, the intervention gets its definite form and crucial choices are made. It would be helpful to collect more evidence on the relationship between methods from theory and practical strategies to support the decision on which strategy to use.

Another core characteristic of IM is the ecological approach [9,12], to ensure that the intended behavior changes are supported by the different layers of the organizational context. The ecological approach considers the complex and layered context in which the intervention is developed and implemented. The included studies all followed the ecological approach by considering behavioral as well as environmental factors on which the interventions were targeted. All interventions contained elements targeted at workers as well as the workplace (e.g., supervisors, physical environment) to accomplish changes in the intended outcomes. However, in the discussion, some authors of the included studies recommend (even) more focus on contextual factors from the beginning of the intervention design to the very end of the implementation [30,38]. This would ensure the feasibility of the intervention and the fit of the intervention within the (changing) organizational context. In addition, the ecological approach could also benefit from more participation of actors from all layers of the organization. Including more actors in the intervention’s development may, however, increase the complexity and costs.

An additional important characteristic of IM is that planning of the intervention implementation is part of the intervention development to decrease the risk of unsuccessful implementation [13]. However, of all the IM steps, the fidelity of the implementation planning (Step 5) was lowest. Almost none of the included studies reported performance objectives for program use. Although the importance of the implementation of interventions is getting more and more attention, in practice for the included studies the development of the intervention design is described in far more detail compared to the planning of the implementation. Almost none of the studies included maintainers in the implementation process. This could be linked to the way these interventions are often financed: by a four-year grant that ends after the evaluation has been completed. However, as some authors of the included studies conclude, by not including plans for maintenance during the intervention design, there is a high risk of the intervention not being maintained after the research project has finished [46]. Fernandez et al. [13] propose implementation mapping as an expansion of the IM intervention planning phase (Step 5) and provide additional details and examples for developing and selecting implementation strategies. Implementation mapping could be used by intervention planners to improve and expand the implementation planning of their interventions.

To summarize, the review of the fidelity of the application of the IM-protocol showed that all included studies had difficulties following the IM-protocol in one way or another. Studies had difficulties following the participative approach, conducting matrices of change objectives, and planning the implementation of the intervention. Practical tools for organizing participation and planning the implementation process (e.g., based on implementation mapping (13)) may help intervention developers to tackle these problems. 

### 4.2. Relation Fidelity IM-Protocol, Implementation and Intervention Effect

There appears to be no clear relation between the fidelity of the IM-protocol and intervention effects. This study found that half of the ORP-HP interventions designed using IM, was effective on primary outcomes, a fourth was partially effective, and a fourth was not effective. Although the IM protocol (Step 6) encourages evaluation on changes in determinants and change objectives, and to explore mediating and moderating variables [9], the effect (and process) evaluation of the included studies often did not include behavioral and environmental determinants as secondary outcomes. Including behavioral and environmental determinants in the evaluations would provide more insights into reasons for (in)effectiveness of interventions and would provide insight into the mechanism of change [57,58].

Subsequently, there appears to be no clear relation between the overall fidelity of the IM-protocol and the implementation process. Regarding the implementation of the interventions, reach appeared to be unsatisfactory in a majority of the studies. Most of the design articles did not elaborate much on the recruitment procedure of participants in the planning phase of the intervention and its implementation. More attention for recruitment during the planning of the implementation could possibly improve the reach of the intervention. Results also show that the fidelity of the implementation process was relatively low. Although high fidelity is considered by many as an important indicator for implementation success, one could question whether a high fidelity actually indicates a successful implementation. Adapting the intervention during implementation in case of changes in the organizational context may often be necessary for a tailored approach. This is supported by a review by Durlak and Dupre [59] that shows that when fidelity does not reach 100%, adaptations could be a positive contribution to outcomes instead of labeling these adaptations as an implementation failure.

Although we found no relation between the overall fidelity of the IM-protocol and the implementation process, there appears to be a relation between the fidelity of the activities related to the theory-based approach (as one of the core elements of IM), and the implementation process, suggesting a high fidelity regarding the theory-based approach, to be related to a more successful implementation (especially to satisfaction and dose received). This may imply that conducting matrices of change objectives, although challenging and time-consuming, could ultimately pay off, resulting in a tailored intervention that matches the target group.

### 4.3. Strengths and Limitations

Several strengths and limitations should be mentioned regarding the design of this study, that may have affected the overall results. First, relatively few intervention studies on ORP and HP have used the IM protocol, resulting in a small selection of (primarily Dutch) studies, making it difficult to quantitatively compare the fidelity of the IM protocol to the implementation process and the intervention effects. 

In line with Fassier et al. [14], an effort was made to systematically identify and review ORP-HP interventions using the IM protocol, and the accompanying studies on implementation and effects. Since a validated protocol to review the studies was not available, the authors developed checklists and followed a structured method to review and rate the IM-steps, implementation process, and intervention effect. However, the selection of included studies consisted of a variety of heterogeneous interventions implemented in different contexts, and the studies differed in relation to the detail that was provided regarding the IM steps, which challenged the standardization of the assessment. Reviewing the process evaluations was particularly challenging. The studies differ regarding the extension of the process evaluation that was conducted. For some of these studies, there was not enough information on the process indicators available to compute mean scores. The studies also differed in relation to the theoretical frameworks on which the process evaluation was based, and some studies did not use a theoretical framework at all, in line with earlier research [60]. Finally, the studies differed regarding the indicators that were taken into account, making comparability of the results of the process evaluation of all the studies challenging. 

It should be noted that the evaluation planning (Step 6) was not part of the review of the fidelity of the IM protocol. However, the evaluation planning (e.g., the study design and timing of measurements) could impact the probability to find effects. For example, to measure intervention effects, the timing of the measures should match the timing of the hypothesized effects on the behavioral outcomes, taking into account the planning of the implementation and anticipating possible barriers during implementation. This information was not included in this study, and therefore we cannot rule out that finding no intervention effects could be due to poor evaluation planning.

Despite the methodological limitations, this study has several strengths as well. To our knowledge, this is the first study that systematically compared the intervention design using IM, to the implementation process as well as the intervention effect. To increase our knowledge on the relationship between intervention design, implementation, and effect, this type of systematic review may provide valuable new insights. It would be even more valuable to link specific behavioral change methods (as part of the intervention design) to behavioral determinants (in an effect evaluation), and to more explicitly link the planning of the implementation (specifying performance objectives for implementation) to process indicators of implementation (in a process evaluation). However, this would request for studies making a standardized reporting of behavior change methods, and consequently studying the effects of behavior changes methods on performance objectives, for the intervention effects as well as the implementation process. This would broaden the evidence base on which behavioral change methods work best to change specific determinants in different contexts.

## 5. Conclusions

Based on the results of this review, some conclusions can be drawn regarding the use of the IM protocol for the development of ORP-HP interventions. The review of the fidelity of the application of the IM-protocol showed that all included studies had difficulties following the IM-protocol in one way or another. Studies had difficulties following the participative approach, conducting matrices of change objectives, and planning the implementation of the intervention. 

Overall, this review did not find a relation between the fidelity of the IM-protocol and the intervention effects. However, results suggest that the implementation process may benefit from a logic model of change as part of the intervention design.

Practical tools for organizing participation, and planning the implementation process (e.g., based on Implementation Mapping [13]) may help intervention developers during intervention development. Simplification or shortening of the IM protocol may also help increase the feasibility of the use of IM. However, this study suggests that the theory based approach, which is considered complex and time-consuming and (for this reason) is often simplified or lacking, can be considered an important part of the intervention development.

## Figures and Tables

**Table 1:** Steps and activities of the intervention mapping (IM) protocol (based on Bartholomew et al. [9]).

**Step 1: Logic model of the problem**
Establish and work with a planning group Conduct a needs assessment to create a logic model of the problem Describe the context for the intervention, including the population, setting, and community State program goals
**Step 2: Program outcomes and objectives; logic model of change**
State expected outcomes for behavior and environment Specify performance objectives for behavioral and environmental outcomes Select determinants for behavioral and environmental outcomes: Construct matrices of change objectives Create a logic model of change
**Step 3: Program design**
Generate program themes, components, scope, and sequence Choose theory and evidence-based change methods Select or design practical applications to deliver change methods
**Step 4: Program Production**
Refine program structure and organization Prepare plans for program materials Draft messages, materials, and protocols Pretest, refine, and produce materials
**Step 5: Program implementation plan**
Identify potential program users (implementers, adopters, and maintainers) State outcomes and performance objectives for program use Construct matrices of change objectives for program use Design implementation interventions
**Step 6: Evaluation plan**
Write effect and process evaluation questions Develop indicators and measures for assessment Specify the evaluation design Complete the evaluation plan

**Table 2:** Summary of fidelity assessment IM, implementation process, and intervention effects.

	Oude Hengel, 2011a; 2012; 2013; 2011b [22,28,29,30]	Coffeng, 2012; 2014a; 2014b; 2013 [21,31,32,33]	Van Berkel, 2011; 2014a; 2014b; 2013 [20,34,35,36]	Oude Hengel, 2014; Van Deurssen, 2014b; 2014a [27,37,38]	Mc Eachan, 2008; 2011, Lawton, 2014 [19,39,40]	Verweij, 2009 2012; 2013; 2011; 2012 [23,41,42,43,44]	Viester, 2012; 2015; 2014 [18,45,46]	Strijk, 2009; 2012, 2013; 2011 [17,47,48,49]	Brosseau, 2007; Parker, 2009 [26,50]	Riphagen 2013a; 2013b [25,51]	Kwak, 2007; 2009; 2010 [16,52,53]	Looijmans, 2011; 2010 [24,54]
IM fidelity *												
**Step 1: Logic model of the problem**												
1a. Formation of linkage group (participation)	−	−	−	−	−	+	−	−	+	−	−	−
1a. Conduct a needs assessment to create a logic model of the problem (theory-based approach)	+	+	+	+	+	+	+	+/−	+	+	+	+
**Step 2: Program outcomes and objectives;** **Logic model of change**												
2a. Construct matrices of change objectives (theory based)	+	−	−	−	+	+	+	−	+	−	−	−
7b. Participative approach (step 1 and/or step 2) (participation)	+	+	+	+	+/−	+	+	+/−	+	+/−	+/−	+
2c. Differentiation between behavioral and environmental factors (ecological approach)	+	+	+	+	+/−	+	+	+/−	+	+	+	+
**Step 3: Program design**												
3a. Choose theory and evidence-based change methods (theory-based approach)	+	+	+	+	+	+	+	+	+	+	+	+
**Step 4: Program production**												
4a. Participative approach (step 3 and/or step 4) (participation)	−	+	+	+/−	+	+	+	−	+	+/−	+/−	+
4b. Worker and workplace component of intervention (ecological approach)	+	+	+	+	+	+	+	+	+	+	+	+
**Step 5: Program implementation plan**												
5a. Identify potential program users (implementers, adopters, and maintainers) (implementation planning)	+/−	+/−	+/−	+/−	+/−	+/−	+/−	+/−	+/−	+/−	+	+/−
5b. State outcomes and performance objectives for program use (implementation planning)	−	−	−	−	+/−	−	+	−	−	−	−	−
5c. Identify drivers and barriers for implementation (implementation planning)	+	+/−	+	+	+	+	+	+	−	−	−	+/−
Design implementation interventions (implementation planning)	+	+	+	+	+	+/−	+	+	+	+	+	+
5d. Participative approach (step 5) (participation)	+	+	+	+	+	−	+	−	−	+	+	+
Implementation process **												
Reach	++	−	−	−	++	n.m.	−	−	n.m.	n.m.	++	n.m.
Dose delivered	++	++	n.m.	+/−	+/−	++	++	++	n.m.	n.m	n.m	++
Dose received	++	+/−	+/−	−	+/−	+/−	+/−	+/−	n.m.	−	+/−	−
Fidelity	+/−	+/−	+/−	+	n.m.	+/−	+/−	+/−	n.m.	n.m.	+/−	n.m.
Satisfaction	+	+	+	+	n.m.	+	+	+	++	+/−	+	n.m.
Intervention effects ***												
Effects	−	+/−	−	+	+	+/−	−	+/−	+ (n.c.)	++	++	+

* IM fidelity rating: + (executed), or +/− (partially executed), or—(not executed (or not measured/described)); ** Implementation process rating: ++ (excellent), + (satisfactory), or +/− (moderate), or—(unsatisfactory); *** Intervention effects rating: ++ (all primary and secondary outcomes effective), + (all primary outcomes effective and secondary outcomes partially or not effective) or +/− (at least one of the primary and/or secondary outcomes effective, but not all primary outcomes effective) or—(all primary and secondary outcomes not effective). Note: n.c.: no control group, n.m.: not measured/not described.

## Supplementary Materials

The following are available online at https://www.mdpi.com/1660-4601/18/4/1775/s1, Table S1: IM fidelity, process implementation, and intervention effect checklist Table S2: Included studies and characteristics of the interventions; Table S3: Data extraction IM fidelity; Table S4a: Data extraction process evaluation study 1–6; Table S4b: Data extraction process evaluation study 7–12; Table S5: Summary of assessment intervention effect; Table S6: Results of the IM fidelity review, implementation process review and effect review translated into scores [used for the figures]; Figure S1: Scatterplot of scores on overall IM fidelity (score 0–2) and implementation process (score 0–3) per study (the dotted lines show the average scores for the overall IM fidelity and the implementation process). For three of the process evaluations, there was not enough data available to calculate a process score (Looijmans, 2010 [54], Riphagen, 2013 [25] and Parker, 2009 [50]); Figure S2: Scatterplot of scores on IM fidelity (score 0–2) and intervention effect (score 0–3) per study (the dotted lines show the average scores for the overall IM fidelity and the effects)..
