# Supplementary material for: Use of Intervention Mapping for Occupational Risk Prevention and Health Promotion: A Systematic Review of Literature
Source: Int J Environ Res Public Health. 2021 Feb 11;18(4):1775. doi: 10.3390/ijerph18041775 (PMC7918071; doi:10.3390/ijerph18041775)
Supplement: Supplementary file 1 [file ijerph-18-01775-s001.pdf]

Supplementary files: Use of intervention mapping for occupational risk prevention and health promotion: a systematic review of literature

Table 1. IM fidelity, process implementation and intervention effect checklist.

**IM characteristics**	**IM steps**	**Rating**<br>**-**	**+/-**	**+**	**++**
	**Step 1: Logic model of the problem**				
Participation	**1a. Formation of planning group**	No formation of a planning group/working group/project group was mentioned		Formation of planning group/working group/project group was mentioned	
Theory - based	**1b.Conduct a needs assessment to create a logic model of the problem**	No needs assessment performed	Needs assessment performed, determinants identified, but no causal pathways mentioned	Needs assessment performed, determinants identified and causal pathways mentioned	
	**Step 2: Program Outcomes and Objectives; Logic Model of Change**				
Theory - based	**2a.Construct matrices of change objectives**	No matrices of change objectives presented		Matrices of change objectives presented	
Participation	**2b.Participative approach (step 1 and/or step 2)**	No participation during Step 1 and/or 2	Participation in Step 1 and/or 2 of target group OR other stakeholders	Participation in Step 1 and/or 2 of target group AND other stakeholders	
Ecological approach	**2c. Differentiation between behavioral and environmental factors**	No behavioral or environmental factors mentioned	Only behavioral factors mentioned, no environmental factors mentioned	Behavioral and environmental factors mentioned	
	**Step 3: Program design**				
Theory - based	**3a.Choose theory- and evidence based change methods**	No theory/evidence based change methods mentioned	Theory and evidence based methods mentioned, BUT no behavioral change theory used	Theory/evidence based change methods mentioned AND behavioral change theory used	
	**Step 4: Program Production**				
Participation	**4a.Participative approach (step 3 and/or step 4)**	No participation/ no participation mentioned	Participation of stakeholders other than target group	Participation of target group	
Ecological approach	**4b. Worker and workplace component of intervention**	Intervention consists of no workplace and no worker component	Intervention consists of workplace OR worker component	Intervention consists of workplace AND worker component	
	**Step 5: Program Implementation Plan**				

Implementation planning	**5a.Identify potential program users: adopters/target group, implementers and maintainers**	Potential program users not explicitly mentioned	Adopters/target group implicitly or explicitly mentioned, implementers OR maintainers explicitly mentioned:	Adopters/target group implicitly or explicitly mentioned, implementers AND maintainers explicitly mentioned	
Implementation planning	**5b.State outcomes and performance objectives for program use**	No target behavior for program use mentioned and not clear if target behavior for program use was determined	Target behavior of adopters or implementors not explicitly mentioned, but likely that target behavior was determined	Target behavior of adopters or implementors explicitly mentioned	
Implementation planning	**5c.Identify drivers and barriers for implementation**	No drivers/barriers for implementation mentioned, and not clear if they have been determined	No drivers/barriers for implementation mentioned, but likely that they have been determined	Drivers/barriers for implementation mentioned	
Implementation planning	**5d.Design implementation interventions**	No implementation interventions mentioned, and not clear if they have been determined	No implementation interventions mentioned, but likely that they have been determined	Implementation interventions mentioned	
Participation	**5e.Participative approach (step 5)**	No participation/ no participation mentioned	Participation of target group or other stakeholders (excluding implementors)	Participation of implementors	
**Process indicators**	**Operationalization**	**-**	**+/-**	**+**	**++**
**Reach (%)**	The proportion of the eligible workers that participates in an intervention (% response to baseline questionnaire or % of signed informed consents)	<50%	50-59%	60-69%	70% or higher
**Dose delivered (mean[a])**	The proportion of intended intervention component delivered or provided.	<60%	60-69% (or major differences between intervention components: from – to ++)	70-79%	80% or higher
**Dose received (mean[a])**	The proportion of participants participating in/using intervention components.	<50%	50-59% (or major differences between intervention components: from – to ++)	60-69%	70% or higher
**Satisfaction (scale 1-10 (%)**	The extent to which the target group of the intervention is satisfied with the intervention	<5 (<50%)	5.0-5.9 (50-59%) (or major differences between intervention components: from – to ++)	6 – 7.9 (60-79%)	8 or higher (80% or higher)
**Fidelity (yes/no)**	The extent to which the intervention was delivered as planned (according to protocol).		adjustments are made to original protocol	no adjustments are made to original protocol	
**Intervention effect**		**-**	**+/-**	**+**	**++**

**Interven-tion effects**		primary and secondary outcomes not effective	primary and/or secondary outcomes partially effective (but primary out-comes not effective)	primary outcomes overall effec-tive, secondary outcomes not or partially effective	All primary and secondary outcomes effec-tive

[a] If dose delivered or dose received was calculated for more than one intervention component, the authors computed a mean of these components.

Table 2. Included studies and characteristics of the interventions.

Authors	Intended outcome	Target group	Sector	Intervention
Oude Hengel, 2011a (de-sign); 2012; 2013 (effect); 2011b (process) (22,28,30)	Work ability (ORP)	Construction workers	Construction sector	Programme objective 1 (restore balance between physical load and need for recovery): protocol for two individual training sessions by a physical thera-pist (including assessment by therapist, 3 personal advices on pocket size card) and a Rest-Break tool.<br>Programme objective 2 (increase range of influence at the worksite): 2 em-powerment training sessions
Coffeng, 2012 (design); 2014a; 2014b (effect); 2013 (process) (21,31-33)	Need for recovery<br>Physical activity<br>Relaxation (ORP and HP)	Office employees	Financial service sec-tor	1. Group Motivational interviewing (GMI) is delivered by teamleaders of the departments allocated to the intervention. They conduct 3 GMI-sessions with employees in their teams, and a booster session. The aim of the session is to stimulate physical activity and relaxation. Teamleaders have 2 GMI-coachingsessies, supervised by a GMI professional to share experiences. The GMI-sessions are supported by a web-based social media platform.<br>2. Environmental modifications: changing coffee corners (add bar with bar chairs), open office environment (exercise balls, curtains to reduce back-ground noise), meeting rooms (standing table and poster) and entrance hall (table tennis, lounge chairs), by creating Active and Relax zones. In addition, footsteps are placed to promote stair walking.
Van Berkel, 2011 (design); 2014a; 2014b (effect); 2013 (process) (20,34-36)	Work engagement<br>Mental health<br>Lifestyle behavior (ORP and HP)	High educated work-ers	Research institutes	Mindfulness training (participatory focus group meetings were held to de-velop mindfulness training program). 8 sessions of 90 minutes by certified trainers. Cognitive components (enjoying here and now, count blessings etc.), behavioral components (home exercise-complementing colleagues), motivational components (goal setting, increasing resilience).<br>Exercise behavior, rest behavior (EBRB) targeted components: exercises in mindfulness training aimed at determinants of EBRB (walking meditation, mindful eating), E-coaching to continue implementation of mindfulness principles learnt in training. Make Personal Energy Plan (PEP), supporting elements (providing fruit, providing routes for lunch walking and stimula-tion to find buddy for several activities.

Oude Hengel, 2014 (design); Van Deurssen, 2014b (effect); 2014a (process) (27,37,38)	Quartz exposure (ORP)	Dutch construction workers and managers	Construction sector	Intervention is called: 'Working Relieved' Baseline: Mailing to workers (invitation, information and feedback).<br>Month 1: Toolbox 1, plenary sessions (video, introduction technical devices and interactive presentation, factsheets, posters).<br>Month 3: Toolbox workers: group sessions at worksite, video, identifying barriers and solutions and tailored advice, assignment. Toolbox employers shared program for the four employers to demonstrate and practice technical control measures.<br>Month 5: personal postcard.<br>Month 6: Toolbox (employer & workers), plenary sessions, providing feedback, discussing assignment and presentation.
Mc Eachan, 2008 (design); 2011 (effect), Lawton, 2014 (process) (19,38,39)	Physical activity (HP)	Employees in sedentary occupation	Variety of sectors	Key components of AME for ACTIVITY intervention: launch week, interactive leaflets, posters, knowledge quiz, setting personal targets, making plans, self-monitoring, team challenges, management support, newsletters and reminders.
Verweij, 2012 (design); 2013 (effect); 2011, 2012 (process) (23,41-44)	Weight gain (HP)	Occupational physicians (to facilitate health promotion activities for employees)	Variety of sectors	The Balance@Work intervention consisted of an occupational health guideline, consisting of three sections<br>a) prevention at the environmental level (advice for employers based on environment scan)<br>b) prevention at the individual level (advice for employees; Ops were trained in an adapted form of motivational interviewing and provided 5 counselling sessions to promote employees' healthy lifestyle; Employees were provided with tools to monitor their behavior), and<br>c) evaluation and maintenance.
Viester, 2012 (design); 2015 (effect); 2014 (process) (18,45,46)	Physical activity & dietary patterns (HP)	Blue collar workers (i.e. construction site and production workers)	Construction sector	The VIP in construction intervention programme consists of tailored information, face-to-face and telephone counselling, exercises and materials designed for the intervention (circumference measuring tape, pedometer, BMI-card, calorie guide, cookbook, knowledge tests, Personal Energy Plan forms) and an overview of the company health promoting facilities.
Strijk, 2009 (design); 2012, 2013 (effect); 2011 (process)(17,47-48)	Lifestyle & vitality (HP)	Older workers (45+) of an academic hospital	Health care sector	The Vital@Work intervention insisting of two parts.<br>1. The Vitality Exercise Programme (VEP): yoga group sessions, workout groups sessions (once a week) and aerobic exercises Free fruit was provided at the guided group sessions of the VEP.<br>2. Three visits to a Personal Vitality Coach (PVC).
Brosseau, 2007 (design); Parker, 2009 (effect/process)(26,50)	Safety (ORP)	Owners and employers of small metal fabrication businesses	Metal industry	Presentation and discussion of report on machine and shop safety audits and employee surveys; presentation and demonstration of compact disc with checklists, tailored programs for lock-out, hazard recognition; list of resources; information about Minnesota OSHA grant process; Placards for 23

				machines; guidelines for a model safety committee; training materials; Further assistance if requested by owner; Building skills and knowledge of a health and safety committee.
Riphagen 2013a (design/process); Riphagen 2013b (effect)(25, 51)	Influenza vaccination (ORP)	Health care workers of University Medical Centers	University Medical Centers	A transparent influenza vaccination intervention implementation strategy, consisting of educational tools, influenza vaccination campaign (website, badges folders, video, posters, information meeting)
Kwak, 2007 (design); 2009 (effect/process); 2010 (effect)(16,52,53)	Weight gain prevention (HP)	Workers	Different sectors	Individual component: feedback on body composition measures, ''in balance-box with self-monitoring devices, website with general information, two CD-ROMs (awareness of weight status and assisting participants with changing WGPBs. Environmental components: handbook serving as guide for the worksite linkage board to assist them through different stages of environmental interventions (e.g. change food assortment, workshops, info wall, prompts for stair use promotion etc.)
Looijmans, 2011 (design); 2010 (effect/process) (24,54)	Influenza vaccination (ORP)	Health care workers	Nursing homes	Three program components: 1. Visit to all nursing homes 2. Plenary information meetings, and 3. Appointment of a program coordinator in each home

Table 3. Data extraction IM fidelity.

	Oude Hengel et al. 2011 (22)	Coffeng et al. 2012 (21)	Van Berkel, 2011 (20)	Oude Hengel, 2012 (27)	McEachan, 2008 (19)	Verweij, 2011 (23)	Viester, 2012 (18)	Strijk, 2009 (17)	Brosseau, 2007 (26)	Riphagen, 2013 (25)	Kwak, 2007 (16)	Looijmans, 2011 (24)
**1a. Formation of planning group** (participation)	Planning group is not explicitly mentioned<br><br>Rating: -	Planning group is not explicitly mentioned<br><br>Rating: -	Planning group is not explicitly mentioned<br><br>Rating: -	Planning group is not explicitly mentioned<br><br>Rating: -	Planning group is not explicitly mentioned<br><br>Rating: -	An expert group was formed consisting of target group and lifestyle experts.<br><br>Rating: +	Planning group is not explicitly mentioned<br><br>Rating: -	Planning group is not explicitly mentioned<br><br>Rating: -	Advisory board which consisted target group and other stakeholders (e.g. policy makers,	Planning group is not explicitly mentioned<br><br>Rating: -	Planning group is not explicitly mentioned<br><br>Rating: -	Planning group is not explicitly mentioned<br><br>Rating: -

	Oude Hengel et al. 2011 (22)	Coffeng et al. 2012 (21)	Van Berkel, 2011 (20)	Oude Hengel, 2012 (27)	McEachan, 2008 (19)	Verweij, 2011 (23)	Viester, 2012 (18)	Strijk, 2009 (17)	Brosseau, 2007 (26)	Riphagen, 2013 (25)	Kwak, 2007 (16)	Looijmans, 2011 (24)
									professional, technical school faculty, union representative) Rating: +			
**1b.Conduct a needs assessment to create a logic model of the problem** (theory based approach)	Needs assessment performed, determinants identified and causal pathways mentioned Rating: +	Needs assessment performed, determinants identified and causal pathways mentioned Rating: +	Needs assessment performed, determinants identified and causal pathways mentioned Rating: +	Needs assessment performed, determinants identified and causal pathways mentioned Rating: +	Needs assessment performed, determinants identified and causal pathways mentioned Rating: +	Needs assessment performed, determinants identified and causal pathways mentioned Rating: +	Needs assessment performed, determinants identified and causal pathways mentioned Rating: +	Needs assessment performed, causal relations are not mentioned literally Rating: +/-	Needs assessment performed, determinants identified and causal pathways mentioned Rating: +	Needs assessment performed, determinants identified and causal pathways mentioned Rating: +	Needs assessment performed, determinants identified and causal pathways mentioned Rating: +	Needs assessment performed, determinants identified and causal pathways mentioned Rating: +
**2a.Construct matrices of change objectives** (theory based approach)	Matrices of change objective were made Rating: +	Matrices of change objective were not made Rating: -	Matrices of change objective were not made Rating: -	Matrices of change objective were not made Rating: -	Matrices of change objective were made Rating: +	Matrices of change objective were made Rating: +	Matrices of change objective were made Rating: +	Matrices of change objective were not made Rating: -	Matrices of change objective were made Rating: +	Matrices of change objective were not made Rating: -	Matrices of change objective were not made Rating: -	Matrices of change objective were not made Rating: -
**2b.Participative approach (step 1**	Round table discussions (target group)	Questionnaires and focus	Interviews (stakeholders_,	Survey (target group) Focus	Focus groups (target group)	Interviews (target group,	Focus group interviews (target	Focus group interviews	Consultation of advisory board	Discussion research team and	Focus groups (not clear	Interviews and focus group ses-

	Oude Hengel et al. 2011 (22)	Coffeng et al. 2012 (21)	Van Berkel, 2011 (20)	Oude Hengel, 2012 (27)	McEachan, 2008 (19)	Verweij, 2011 (23)	Viester, 2012 (18)	Strijk, 2009 (17)	Brosseau, 2007 (26)	Riphagen, 2013 (25)	Kwak, 2007 (16)	Looijmans, 2011 (24)
**and/or step 2)** (participation)	& interviews HRM (stakeholders)<br><br>Rating: +	group interviews (target group), interviews (stakeholders).<br><br>Rating: +	questionnaire and focus groups (target group)<br><br>Rating: +	groups and interviews (stakeholders)<br><br>Rating: +	Rating: +/-	stakeholders)<br><br>Rating: +	group, stakeholders)<br><br>Rating: +	(target group)<br><br>Rating: +/-	(representation of target group and other stakeholders)<br><br>Rating: +	other stakeholders. Not clear if target group was involved directly.<br><br>Rating: +/-	with whom)<br><br>Rating: +/-	sions (target group and stakeholders)<br><br>Rating: +
**2c. Differentiation between behavioral and environmental factors** (ecological approach)	Personal and environmental determinant are mentioned<br><br>Rating: +	Personal and environmental determinant are mentioned<br><br>Rating: +	Personal and environmental determinant are mentioned<br><br>Rating: +	Behavioural and organizational determinant are mentioned<br><br>Rating: +	Behavioural determinants are mentioned<br><br>Rating: +/-	Personal and environmental determinant are mentioned<br><br>Rating: +	Personal and environmental determinant are mentioned<br><br>Rating: +	In this stage environmental determinants/ factors not mentioned.<br><br>In step 3, methods involved environmental changes.<br><br>Rating: +/-	Personal and environmental determinant are mentioned<br><br>Rating: +	Personal and environmental determinant are mentioned<br><br>Rating: +	Personal and environmental determinant are mentioned<br><br>Rating: +	Behavioural organisational and demographical determinants are mentioned<br><br>Rating: +
**3a.Choose theory- and evidence based change methods**	Table with theory based methods<br><br>Rating: +	Table with theory based methods<br><br>Rating: +	Table with theory based methods<br><br>Rating: +	Table with theory based methods<br><br>Rating: +	Table with theory based methods Literature review to determine	Table with theory based methods<br><br>Rating: +	Table with theory based methods<br><br>Rating: +	Table with theory based methods<br><br>Rating: +	Theory based methods are mentioned in the text	Theory based methods are mentioned in the text	Table with theory based methods<br><br>Rating: +	Table with theory based methods<br><br>Rating: +

	Oude Hengel et al. 2011 (22)	Coffeng et al. 2012 (21)	Van Berkel, 2011 (20)	Oude Hengel, 2012 (27)	McEachan, 2008 (19)	Verweij, 2011 (23)	Viester, 2012 (18)	Strijk, 2009 (17)	Brosseau, 2007 (26)	Riphagen, 2013 (25)	Kwak, 2007 (16)	Looijmans, 2011 (24)
(theory based approach)					most effective strategies<br><br>Rating: +				Rating: +	Rating: +		
**4a.Participative approach (step 3 and/or step 4)** (participation)	Not mentioned in this stage<br><br>Rating: -	Strategies based on focus group (target group) advice of project group, and feedback from experts in the field (other stakeholders)<br><br>Rating: +	Focus groups (target group) to develop mindfulness program<br><br>Rating: +	First version was subjected to commentary by researchers, managers and sector organisations (stakeholders)<br><br>Rating: +/-	Focus group (target group), expert steering group, contacts within participating organisations(stakeholders)<br><br>Rating: +	Interview data (target group) Experts in the field of lifestyle commented on first draft<br><br>Rating: +	Feedback from key contacts within the organization (stakeholders) and focus group data (target group)<br><br>Rating: +	Not mentioned in this stage<br><br>Rating: -	Input from advisory board (stakeholders). Pilot test with target group<br><br>Rating: +	Collaboration with UMC (stakeholders)<br><br>Rating: +/-	Brainstorm session with experts.<br><br>Rating: +/-	Collaboration with UMC, pretested by target group<br><br>Rating: +
**4b. Worker and workplace component of intervention** (ecological approach)	Components target personal and environmental determinant<br><br>Rating: +	Components target individual behaviour and physical environment<br><br>Rating: +	Components target individual behaviour and physical environment<br><br>Rating: +	Intervention components are aimed at workers and managers, materials are made available.	Intervention components are aimed at awareness, motivation and environment.<br><br>Rating: +	Intervention components are aimed at individual behaviour and environment<br><br>Rating: +	Intervention components are aimed at personal and external determinant<br><br>Rating: +	Intervention components are aimed behavioural determinants, and require some en-	Intervention program was targeted at employees and business owners.<br><br>Rating: +	Intervention components are aimed at personal and external determinants<br><br>Rating: +	The intervention was arranged into two components, an individual component and a worksite	Methods and strategies were aimed at management and HCW level<br><br>Rating: +

	Oude Hengel et al. 2011 (22)	Coffeng et al. 2012 (21)	Van Berkel, 2011 (20)	Oude Hengel, 2012 (27)	McEachan, 2008 (19)	Verweij, 2011 (23)	Viester, 2012 (18)	Strijk, 2009 (17)	Brosseau, 2007 (26)	Riphagen, 2013 (25)	Kwak, 2007 (16)	Looijmans, 2011 (24)
				Rating: +				vironmental changes<br><br>Rating: +			component<br><br>Rating: +	
**5a.Identify potential program users: adopters/ target group, implementers and maintainers** (implementation planning)	Adopters/ target group and implementers are mentioned<br><br>Rating: +/-	Adopters/ target group and implementers are mentioned<br><br>Rating: +/-	Adopters/ target group and implementers are mentioned<br><br>Rating: +/-	Adopters/ target group and implementers are mentioned<br><br>Rating: +/-	Adopters/ target group and implementers are mentioned<br><br>Rating: +/-	Adopters/ target group and implementers are mentioned<br><br>Rating: +/-	Adopters/ target group and implementers are mentioned<br><br>Rating: +/-	Adopters/ target group and implementers are mentioned<br><br>Rating: +/-	Adopters/ target group and implementers are mentioned<br><br>Rating: +/-	Adopters/ target group and implementers are mentioned<br><br>Rating: +/-	Adopters/ target group, implementers and maintainers are mentioned<br><br>Rating: +	Adopters/ target group and implementers are mentioned<br><br>Rating: +/-
**5b.State outcomes and performance objectives for program use** (implementation planning)	Not mentioned<br><br>Rating: -	Not mentioned<br><br>Rating: -	Not mentioned<br><br>Rating: -	Not mentioned<br><br>Rating: -	Target behavior of adopters or implementors not explicitly mentioned, but likely that target behavior was determined:	Not mentioned<br><br>Rating: -	Target behavior of adopters or implementors explicitly mentioned<br><br>Rating: +	Not mentioned<br><br>Rating: -	Not mentioned<br><br>Rating: -	Not mentioned<br><br>Rating: -	Not mentioned<br><br>Rating: -	Not mentioned<br><br>Rating: -

	Oude Hengel et al. 2011 (22)	Coffeng et al. 2012 (21)	Van Berkel, 2011 (20)	Oude Hengel, 2012 (27)	McEachan, 2008 (19)	Verweij, 2011 (23)	Viester, 2012 (18)	Strijk, 2009 (17)	Brosseau, 2007 (26)	Riphagen, 2013 (25)	Kwak, 2007 (16)	Looijmans, 2011 (24)
					"Step 5 then, essentially involved repeating steps 2-3 of the intervention mapping process for behaviours we specifically wished to see from the facilitators"<br><br>Rating: +/-							
**5c.Identify drivers and barriers for implementation** (implementation planning)	Several barriers for the intervention are mentioned<br><br>Rating: +	Strengths and limitations are not literally mentioned in the paper, but discussion about strengths and limitations had taken	Possibilities for success and potential challenges for implementation were discussed<br><br>Rating: +	Drivers and barriers for adoption were mentioned<br><br>Rating: +	Drivers and barriers for adoption were mentioned<br><br>Rating: +	Barriers for adoption were mentioned<br><br>Rating: +	Managers and users were interviewed to gain insight into facilitating factors and barriers<br><br>Rating: +	Drivers and barriers for adoption were mentioned<br><br>Rating: +	not mentioned<br><br>Rating: -	Not mentioned<br><br>Rating: -	not mentioned<br><br>Rating: -	Not mentioned literally, but stakeholders were approached to give feedback on and to support the program. Feedback

	Oude Hengel et al. 2011 (22)	Coffeng et al. 2012 (21)	Van Berkel, 2011 (20)	Oude Hengel, 2012 (27)	McEachan, 2008 (19)	Verweij, 2011 (23)	Viester, 2012 (18)	Strijk, 2009 (17)	Brosseau, 2007 (26)	Riphagen, 2013 (25)	Kwak, 2007 (16)	Looijmans, 2011 (24)
		place and resulted in adaptations.<br><br>Rating: +/-										was used to fine-tune program elements<br><br>Rating: +/-
**5d.Design implementation interventions** (implementation planning)	Measures were taken to optimize adoption/ implementation<br><br>Rating: +	Measures were taken to optimize adoption/ implementation<br><br>Rating: +	Measures were taken to optimize adoption/ implementation<br><br>Rating: +	Measures were taken to optimize adoption/ implementation<br><br>Rating: +	Measures were taken to optimize adoption/ implementation<br><br>Rating: +	No concrete measures are described except a passage in the OP guideline to emphasize confidentiality and resolve resistance from employees<br><br>Rating: +/-	Measures were taken to optimize adoption/ implementation<br><br>Rating: +	Measures were taken to optimize adoption/ implementation<br><br>Rating: +	Measures were taken to optimize adoption/ implementation<br><br>Rating: +	Measures were taken to optimize adoption/ implementation<br><br>Rating: +	Measures were taken to optimize adoption/ implementation<br><br>Rating: +	Measures were taken to optimize adoption/ implementation<br><br>Rating: +
**5e.Participative approach (step 5)** (participation)	Implementation plan was written together with implementers (implementers)	Test with intended users (target group and implementers)<br>Rating: +	Focus group meetings to design implementation plan (implementers)	Meeting with managers to discuss barriers and solutions (implementers)	Focus groups to discuss barriers and solutions (target group	Not mentioned<br><br>Rating: -	Interviews with potential users (target group) HRM involved in program	Not mentioned<br><br>Rating: -	Not mentioned<br><br>Rating: -	UMC contacts and communication staff were visited (implementers)	Linkage board with research team, potential users and implementers	Stakeholders were approached to provide feedback and support the program

	Oude Hengel et al. 2011 (22)	Coffeng et al. 2012 (21)	Van Berkel, 2011 (20)	Oude Hengel, 2012 (27)	McEachan, 2008 (19)	Verweij, 2011 (23)	Viester, 2012 (18)	Strijk, 2009 (17)	Brosseau, 2007 (26)	Riphagen, 2013 (25)	Kwak, 2007 (16)	Looijmans, 2011 (24)
	Rating: +		Rating: +	Rating: +	and implementers)<br><br>Rating: +		development (implementers)<br><br>Rating: +			Rating: +	Rating: +	(stakeholders and implementers)<br><br>Rating: +

Table 4. a: Data extraction process evaluation study 1-6.

Author	Oude Hengel, 2011b (30)	Coffeng, 2013 (33)	Van Berkel, 2013 (36)	Van Deursen, 2014a (38)	Lawton, 2014 (40)	Verweij, 2011,2012 (43,44)
Process evaluation framework	Linnan & Steckler	Linnan & Steckler	Linnan & Steckler & RE-AIM	Linnan & Steckler	No framework, but following similar approach as Dane and Schneider, 1998	Linnan & Steckler
Methods	Questionnaires, logs and checklists, interviews	Registration, program records, observations, questionnaire	data from questionnaires, and semi-structured interviews with participants (high and low compliers wre selected to maximize variety of views) Reach (Glasgow et al)	Logs, company records, checklists, attendance registration forms, questionnaire	Different data sources (minutes, logs, observations, follow-up surveys, archives, study records)	Questionnaires (two levels: Ops and employees)
Reach	85% response baseline questionnaire (293/347)<br>(293 workers, of which 171 in intervention group)<br><br>Rating:++	35% response rate (412/1182)<br><br>Rating: -	14% response rate (257/1820)<br><br>Rating: -	29% response rate (116/404)<br><br>Rating: -	99% response baseline questionnaire<br><br>Rating:++	N.M.
Dose delivered	90-100%<br><br>Rating:++	88%-92% 'acceptable'<br><br>Rating: ++	N.M.	sessions: >95%<br>worksite visits: 20% relatively low<br><br>Rating: +/-	Adherence:<br>Local council: 81% (7,3/9)<br>Hospital: 84% (7,6/9)<br>Bus company: 28% (2,5/9)<br>Government organization: 84% (7,6/9)<br>University: 56% (5/9)<br><br>Rating :+/-	86% of counseling sessions<br><br>Rating: ++
Dose received (Participation intervention component-(range)	63%-79% ('*satisfactory*')<br><br>Session therapist 1: 74%<br>Empowerment training 1: 79%<br>Session therapist 1: 63%<br>Empowerment training 1: 73%<br><br>Rating:++	45-67%<br><br>Rating: +/-	At least once: 30%-81%<br>High compliant (75% of intended use): 6%-54% ('*compliance to training was high, but to e-coaching low*')<br><br>Rating: +/-	28%-54% ('*lower than expected*')<br><br>Session 1: 28%<br>Worksite visit: 20%<br>Session 2: 54%<br><br>At least one: 58%<br>All: 11%<br><br>Rating: -	Received according to questionnaires by workers<br>Exposure to intervention components:<br>Local council: 78% (7,1/9)<br>Hospital: 76% (6,9/9)<br>Bus company: 29% (2,6/9)<br>Government organization: 78% (7/9)<br>University: 27% (3/9)<br><br>Rating: +/-	20%-72%<br><br>72% counselling session<br>60% read flyer<br>42% used measure tape<br>34% used pedo meter<br>20% used diary<br><br>Rating: +/-

**Fidelity**	Modifications had to be made, rest-break tool not implemented as intended<br>Rating: +/-	Improvements had to be made regarding the physical environment to improve implementation<br><br>Rating: +/-	Differences between trainers in how they dealt with buddy system/home work. Rooms were not well equipped.<br><br>Rating: +/-	Compared to previous studies fidelity was high, only the 2nd session was not implemented as intended since the assignment was not completed by construction workers. All other parts were implemented according to protocol<br><br>Rating: +	N.M.	Guideline was partly implemented by OPS as intended. Guideline adherence was assessed as moderate<br><br>Rating: +/-
**Satisfaction**	64% recommends implementation. 'Content was rated moderate.'<br><br>Rating: +	Social environment intervention: 6.0<br>Physical environment intervention: 7.0<br><br>Rating: +	Overall intervention: 7.0<br>Training: 7.9<br>E-coaching: 6.8<br>Fruit: 7.9<br><br>Rating: +	Overall intervention: 7.5 (workers)<br>6.5 (managers)<br><br>Rating: +	N.M.	Workers rated Balance@Work intervention: 7.1<br><br>Rating: +

Note: Implementation process rating: ++ (excellent), + (satisfactory) or +/- (moderate) or – (unsatisfactory).

Table 4. b: Data extraction process evaluation study 7-12.

Author	Viester, 2014b (46)	Strijk, 2011 (49)	Parker, 2009 (50)	Riphagen, 2014a (25)	Kwak, 2009 (52)	Looijmans, 2011 (54)
Process evaluation framework	RE-AIM	Linnan & Steckler	No framework	No framework	Rogers' diffusion theory	Compliance
Methods	web based registration system, follow-up questionnaire, interviews	Attendance registration forms, follow up questionnaire	Process evaluation survey	Qualitative (checklist, annual communication reports) and quantitative (web based questionnaire) process evaluation	two post-test questionnaires, observations and registrations of activities	Registration of visits to nursing homes, questionnaire
Reach	1021 workers invited, 314 included in the study (31%)<br><br>Rating: -	3756 invited, 730 workers were included as they completed baseline (19%)<br><br>Rating: -	N.M.	N.M.	Response to baseline questionnaire: 88% (487/553)<br><br>Rating: ++	N.M.
Dose delivered	coaching appointment: 98%<br>materials: 99%<br>('satisfactory')<br><br>Rating: ++	Yoga session: 72%<br>Work out session: 96%<br>PVC visits: 100%<br>('as planned')<br><br>Rating: ++	N.M.	N.M.	N.M.	All intervention homes were visited and received the materials, all homes organized information meetings<br><br>Rating: ++
Dose received<br><br>(Participation intervention component - range)	15%-61%<br><br>All coaching sessions: 61%<br>Using forms: 26%<br>Pedometer: 52%<br>Measuring tape: 43%<br>BMI card: 30%<br>Calorie card/ cookbook: 15%<br>Exercise card: 62%<br>84% (at least one coaching sessions)<br><br>Rating: +/-	52% attendance rate yoga sessions<br>45% attendance rate work out sessions<br>58% attendance rate PVC visits<br><br>Rating: +/-	N.M.	2009/2020: attendance rate to information session: 24%<br><br>2010/2011 attendance rate to information session: 9%<br><br>Rating: -	5%-87%<br><br>Read personal feedback: 87%<br>Website visit: 75%<br>Carry out advise (energy balance): 21%<br>Carry out advice (physical activity): 29%<br>Take stairs: 50%<br>Take bike: 5%<br><br>Rating: +/-	attendance rate to information session: 7%<br><br>At nursing home level we found a variation in compliance<br>with the intervention<br><br>Rating: -
Fidelity	Adjustments to the program should be made to improve fidelity; 'fidelity was moderate'<br><br>Rating: +/-	'The intervention protocol with respect to the time schedule of the yoga and work out sessions were partly followed by the providers'	N.M.	N.M.	Only two out of six workplaces formed a linkage group<br><br>Rating: +/-	N.M.

		Rating: +/-				
**Satisfaction**	Intervention: 7.6<br><br>Rating: +	Yoga: 7.5<br>Work out: 7.7<br>PVC visits: 6.9<br><br>Rating: +	Program helped improve knowledge: 94%<br>Program improved safety practice: 76%<br><br>Rating: ++	2009/2010:<br>Rated as appealing (1-10):<br>- Badge: 3.2<br>- Poster:9.6<br>- Folder:9.2<br>- Video: 2.8<br><br>Rating: +/-	Intervention components were rated interesting by: 58%-65% and comprehen-sive by: 79%-89%<br><br>Rating: +	N.M.

Note: Implementation process rating: ++ (excellent), + (satisfactory) or +/- (moderate) or – (unsatisfactory).

Table 5. Summary of assessment intervention effect.

Author	Study design	Sample size	Primary outcomes	Secondary outcomes	Rating
Oude Hengel, 2012; 2013 (28,29)	RCT	n=297	Work ability: -<br>Physical and mental health status: -<br>Musculoskeletal symptoms: -<br>Long term sickness absence: -	work engagement: -<br>social support: -<br>Physical workload: -<br>need for recovery: -	-
Coffeng, 2012a; 2014b (31,32)	2X2 factorial study design	n=412	Presenteeism: -<br>Absenteeism,: -<br>Contextual performance: + (opposite direction ; combined intervention)<br>Dedication: + (opposite direction; combined intervention)<br>Task performance: + (social environmental intervention)<br>Absorption: + (physical environmental intervention)	need for recovery: -<br>exhaustion: + (combined intervention)<br>vigorous physical activities: + (combined intervention)<br>small breaks: + (combined intervention)<br>active commuting: + (combined intervention/ physical environmental intervention)<br>exhaustion: + (social environmental intervention)<br>sedentary behavior: + (social environmental intervention/ physical environmental intervention))<br>small breaks: + (social environmental intervention)<br>leisure activities: + (social environmental intervention)<br>stair climbing: + (physical environmental intervention)	+/-
Van Berkel, 2014; 2015 (34,35)	RCT	n=257	work engagement: -<br>mental health: -<br>need for recovery: -<br>mindfulness: -	vigorous physical activity in leisure time: -<br>sedentary behavior: -<br>fruit and vegetable intake: -<br>behavioral determinants: -	-
Van Deursen, 2014b (37)	RCT	n=282	quartz exposure: +	Use of technical control measures: +/- (only effect for subgroup)	+
McEachan, 2011 (39)	RCT	n=1260	systolic blood pressure: +<br>resting heart rate: +<br>body mass index: + (opposite direction)	minutes of activity: -<br>*Subgroup analyses: association between intervention participation and weight gain prevention*	+
Verweij, 2013 (42)	RCT	n=523	body weight: -<br>body weight related outcomes: -<br>CVD-risk factors: -<br>Quality of life: -	sedentary behavior at work: +<br>fruit intake: +<br>physical activity: -<br>sedentary behavior in leisure time: -<br>snack intake: -	+/-
Viester, 2014a (45)	RCT	n=314	musculoskeletal symptoms: -<br>physical functioning: -<br>work ability: -<br>work-related vitality: -<br>work performance: -<br>sickness absence: -		-

Strijk, 2012, 2013 (47,48)	RCT	n=730	vitality: -<br>work engagement: -<br>work performance: -<br>sick leave: -	sport activities: +<br>fruit intake: +<br>Need for recovary: +<br>Vigorous intensity physical activities: -<br>earobic capacity: -<br>mental health: -<br>*subgroup analyses: favourable effects on vitality among high yoga compliers*	+/-
Parker, 2009 (50)	RCT – no con-trol group	n=40	devices or adequate guarding of machine safety: +<br>presence of required safety programs and practices: +<br><br>Difference between T0 and T1 is significant for both outcomes.		+ (no control group)
Riphagen 2014b (51)	Pragmatic RCT	n=3367	Vaccination uptake: +	Nosocomial influenza and/ or pneumonia among health care work-ers: +<br>In-hospital patient morbidity: +	++
Kwak, 2009, 2010 (52,53)	quasi-experi-mental pre-test multiple post control group design	n=487	Skinfold: +<br>Waist circumference: +	Dietary intake: +<br>Physical activity: +<br>Motivational stage: +<br>Cognitive determinants: -	++
Looijmans, 2011 (54)	RCT	n=6636	Vaccination uptake: +<br>*Higher compliance with program elements was associated with higher vaccine uptake.*		+

Note: Intervention effects rating: ++ (all primary and secondary outcomes overall effective), + (all primary outcomes overall effective and secondary outcomes partially or not effective) or +/- (at least one of the primary and/or secondary outcomes partially effective, but not all primary outcomes effective) or – (all primary and secondary outcomes not effective).

Table 6. Results of the IM fidelity review, implementation process review and effect review translated into scores [used for the figures].

	1<br>Oude Hengel et al. 2011 (22)	2<br>Coffeng et al. 2012 (21)	3<br>van Ber-kel, 2011 (20)	4<br>Oude Hengel et al. 2014 (27)	5<br>McEacha n,2008 (19)	6<br>Verweij, 2012 (23)	7<br>Viester, 2012 (18)	8<br>Strijk, 2009 (17)	9<br>Brosseau, 2007 (26)	10<br>Riphagen, 2014 (25)	11<br>Kwak, 2007 (16)	12<br>Looijmans , 2010 (24)	Total
IM Fidelity score (Step 1-5)(score ranged from 0-2)	1,5	1,4	1,5	1,4	1,5	1,5	1,8	0,9	1,5	1,2	1,2	1,4	1,4
Participation	1,0	1,5	1,5	1,3	1,3	1,5	1,5	0,3	1,5	1,0	1,0	1,5	1,2
Theory-based approach	2,0	1,3	1,3	1,3	2,0	2,0	2,0	1,0	2,0	1,3	1,3	1,3	1,6
Ecological model	2,0	2,0	2,0	2,0	1,5	2,0	2,0	1,5	2,0	2,0	2,0	2,0	1,9
Implementation	1,3	1,0	1,3	1,3	1,5	1,0	1,8	1,3	0,8	0,8	1,0	1,0	1,1
Implementation process (score ranged from 0-3)	2,4	1,4	1	1	1,67	1,75	1,4	1,4	-	-	1,75	-	1,5
Reach	3	0	0	0	3	-	0	0	-	-	3	-	1,1
Dose delivered	3	3	-	1	1	3	3	3	-	-	-	3	2,5
Dose received	3	1	1	0	1	1	1	1	-	0	1	0	0,6
Fidelity	1	1	1	2	-	1	1	1	-	-	1	-	1,1
Satisfaction	2	2	2	2	-	2	2	2	3	1	2	-	2
Intervention effect (score ranged from 0-3)	0	1	0	2	2	1	0	1	2	3	3	2	1,2

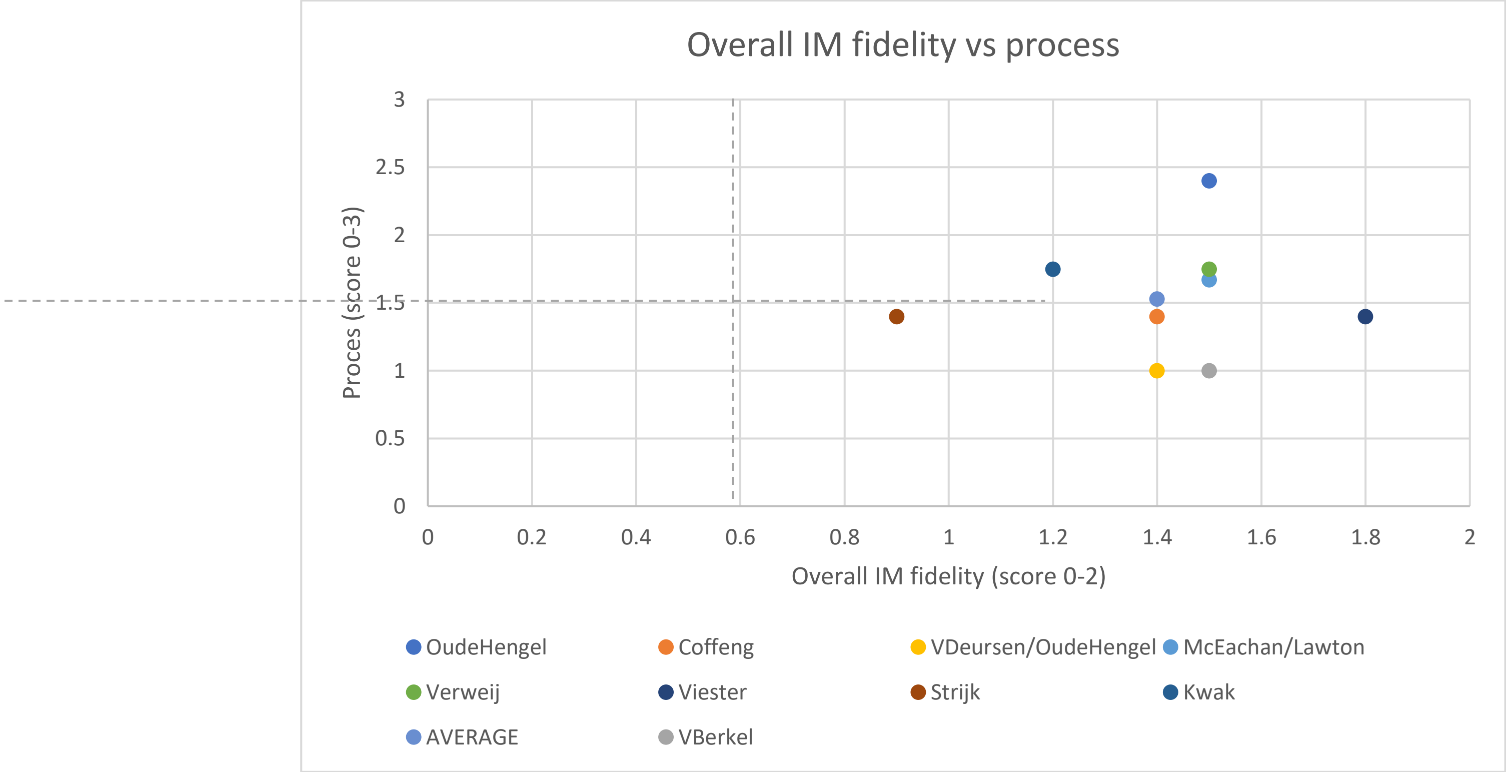

Figure 1. Scatterplot of scores on overall IM fidelity (score 0-2) and implementation process (score 0-3) per study (the dotted lines show the average scores for the overall IM fidelity and the implementation process). For three of the process evaluations, there was not enough data available to calculate a process score (Looijmans, 2010 (54), Riphagen, 2013 (25) and Parker, 2009 (50)).

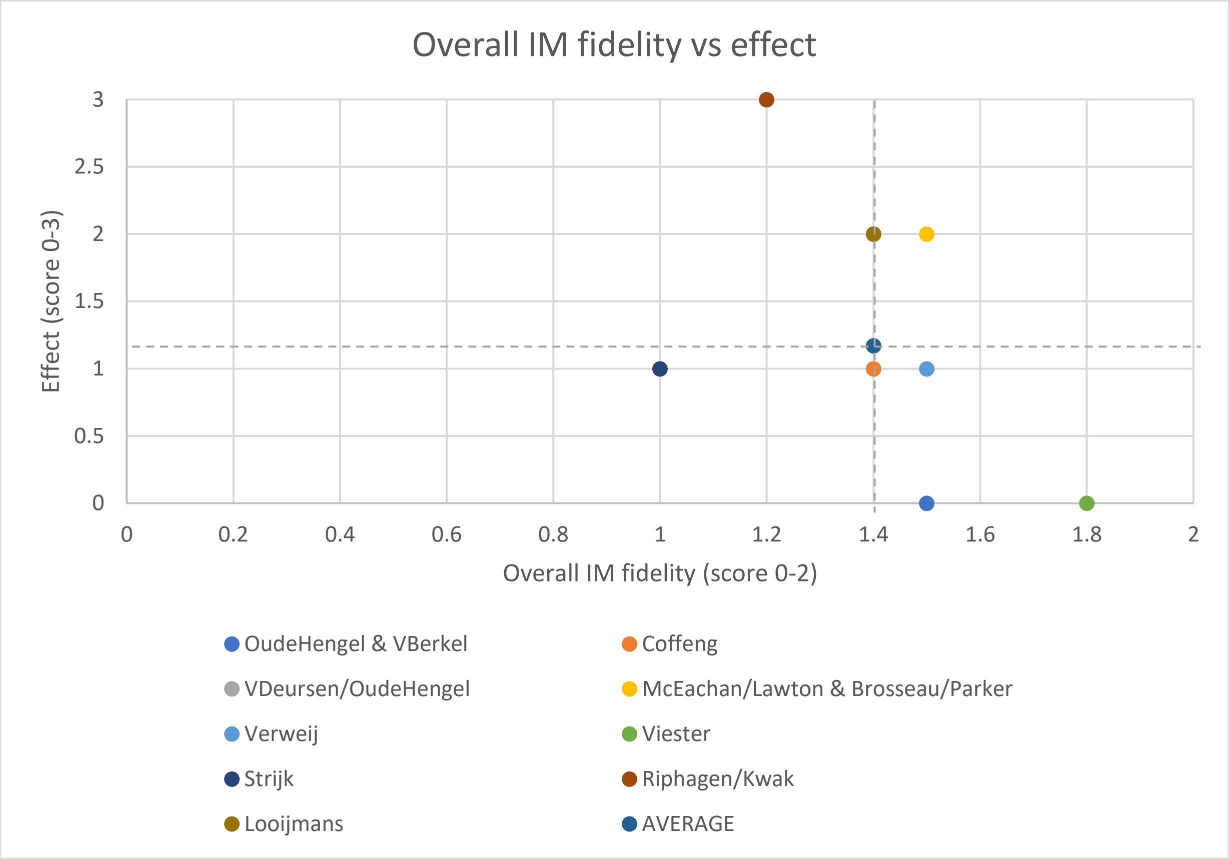

Figure 2. Scatterplot of scores on IM fidelity (score 0-2) and intervention effect (score 0-3) per study (the dotted lines show the average scores for the overall IM fidelity and the effects).
